# The Cry48Aa-Cry49Aa binary toxin from *Bacillus sphaericus* exhibits highly restricted target specificity

**DOI:** 10.1111/j.1462-2920.2008.01667.x

**Published:** 2008-09

**Authors:** Gareth W Jones, Margaret C Wirth, Rose G Monnerat, Colin Berry

**Affiliations:** 1 Cardiff School of Biosciences, Cardiff UniversityMuseum Avenue, Cardiff CF10 3US, UK; 2 Department of Entomology, University of CaliforniaRiverside, California, 92521, USA; 3 Embrapa Recursos Geneticos e Biotecnologia, Parque Estação BiológicaAvenue W/.5 Norte (final), CEP 70 770-900, Caixa Postal 02372 Brasília – DF, Brazil

## Abstract

The Cry48Aa/Cry49Aa binary toxin of *Bacillus sphaericus* was recently discovered by its ability to kill *Culex quinquefasciatus* mosquito larvae through a novel interaction between its two components. We have investigated the target specificity of this toxin and show it to be non-toxic to coleopteran, lepidopteran and other dipteran insects, including closely related *Aedes* and *Anopheles* mosquitoes. This represents an unusually restricted target range for crystal toxins from either *B. sphaericus* or *Bacillus thuringiensis.* Gut extracts from *Culex* and *Aedes* larvae show differential processing of the Cry48Aa protein, with the location of cleavage sites in *Culex* reflecting those previously shown for the activation of Cry4 toxins in mosquitoes. Pre-activation of Cry48Aa/Cry49Aa with *Culex* extracts, however, fails to induce toxicity to *Aedes* larvae. Co-administration of Cry49Aa with Cry4Aa gives higher than predicted toxicity, perhaps suggesting weak synergism against *Culex* larvae between Cry49Aa and other three-domain Cry toxins.

## Introduction

We have recently described a previously unknown toxin from *Bacillus sphaericus* strain IAB59 that is composed of two proteins, Cry48Aa and Cry49Aa (Jones *et al*., 2007). Cry49Aa forms part of a family with the BinA and BinB components of the mosquitocidal binary toxin of *B. sphaericus*, along with their relatives from *Bacillus thuringiensis*, Cry36 (Rupar *et al*., 2003) and Cry35, which itself is part of a binary toxin with the approximately 14 kDa Cry34 (Ellis *et al*., 2002; Baum *et al*., 2004)*.* Cry48Aa is clearly a member of the family of three-domain Cry toxins of *B. thuringiensis* and shows approximately 30% identity with known mosquitocidal toxins such as Cry4Aa. Despite this, neither Cry48Aa nor Cry49Aa are toxic when fed individually to *Culex quinquefasciatus* mosquitoes. However, high-level toxicity to this insect is achieved when the individual proteins are co-administered at the optimum 1:1 ratio (with an LC50 that equates to 15.9 ng ml^−1^ Cry48Aa and 6.3 ng ml^−1^ Cry49Aa (Jones *et al*., 2007)). Cry48Aa/Cry49Aa therefore represent a new insecticidal combination that exploits an interaction between two previously un-associated toxin families. In this study, we investigate the insect target range of this toxin and demonstrate an apparent restriction of toxicity to the *Culex pipiens* complex of mosquitoes. We also investigate possible interactions between Cry49Aa and another related three-domain toxin, Cry4A.

## Results

### Bioassays

In bioassays using the individual recombinant *B. thuringiensis* producing Cry48Aa or Cry49Aa, no toxicity against *Cx. quinquefasciatus* was observed. However, when a combination of the two recombinants was used, 100% larval death occurred, confirming our previous findings (Jones *et al*., 2007). The Cry49Aa protein is a member of a family of proteins that includes Cry36A, which is reported to be somewhat toxic to coleopteran larvae (Rupar *et al*., 2003). However, the Cry48Aa- and Cry49Aa-producing strains showed no toxicity (individually or in combination) to the coleopteran *Anthonomus grandis.* Nor was any toxicity seen when cultures were assayed against the three lepidopteran targets *Anticarsia gemmatalis*, *Spodoptera frugiperda* or *Plutella xylostella.* In all cases, 100% mortality of target insects could be achieved by use of equivalent doses of appropriate *B. thuringiensis* strains (*B. thuringiensis* ssp. *israelensis* for *Diptera*; *B. thuringiensis* ssp. *kurstaki* HD-1 for *Lepidoptera*; *B. thuringiensis* ssp. *tenebrionis* T08 017 for *Anth. grandis*).

The genus *Culex* is in the taxonomic order *Diptera*, suborder *Nematocera*, so toxicity to more closely related insects from this suborder was tested. *Chironomus riparius* larvae were also refractory to the action of the toxins. Of particular note, however, was the complete absence of toxicity of the Cry48Aa and Cry49Aa toxins, individually or as a combination, to the mosquito species *Aedes aegypti* and *Anopheles gambiae* that share the same taxonomic family (*Culicidae*) with *Culex.*

### Toxin synergism

As Cry48Aa is closely related to the Cry4 toxins but seems to require the presence of Cry49Aa for activity against *Cx. quinquefasciatus*, it was of interest to conduct a preliminary assessment of the potential of Cry49Aa to synergize with a Cry4 protein. In bioassays against the Syn-P colony, Cry49Aa crystals, as expected, produced no mortality at up to 200 μg ml^−1^. In contrast, Cry4Aa caused mortality that exhibited a fluctuating plateau from 5 to 200 μg ml^−1^, averaging 59.6%, similar to previous assays with this material against other *Cx. quinquefasciatus* colonies (Wirth and Georghiou, 1997). This behaviour indicates that the Cry4Aa is poorly active and does not fit the assumption of a normal distribution, hence precluding Probit analysis. The Cry4Aa preparation was then co-administered in a 3:1 ratio with Cry49Aa crystals. At 200 μg ml^−1^ (150 μg ml^−1^ Cry4Aa plus 50 μg ml^−1^ Cry49Aa), the observed mortality was 81.3% (standard deviation, sigma = 7.5), 1.7 times higher than the 47% (sigma = 11.5) seen for 150 μg of Cry4Aa alone. Although these preliminary assays do not establish that this apparent increase in activity in the presence of Cry49Aa is statistically significant, the consistently higher mortality caused by the mixture may be indicative of a weak synergistic interaction. However, synergism was not evident at combined toxin concentrations of 20 or 2 μg ml^−1^ and further experiments would be required to definitively establish synergy. Mortality following exposure to Cry4Aa (150 μg ml^−1^) plus Cry49Aa (50 μg ml^−1^) in the Cry4A + Cry4B-resistant colony Cq4AB was 25%. This is again higher than predicted for the mixture, but cannot be statistically distinguished from it because of the high standard deviation (sigma = 18).

### Molecular model of Cry48Aa

blast database searches (Altschul *et al*., 1990) and amino acid sequence analysis of Cry48Aa have revealed that it shows homology to the three-domain Cry toxins for which crystal structures are available. The homology-modelling server, swiss-model (Schwede *et al*., 2003), was used to generate a first-approximation three-dimensional model of Cry48Aa.

**Fig. 1:**
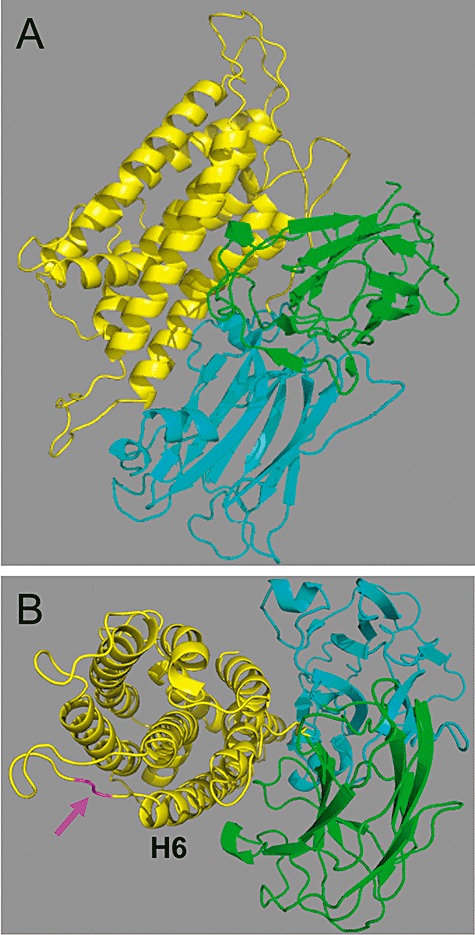
Molecular model of Cry48Aa. Model of Cry48Aa showing domain I (yellow), domain II (blue), domain III (green). The cleavage site for *Culex* gut extracts between the central helices 5 and 6 (H6) is shown in magenta in B. Image created using Pymol software.

While care must be taken when considering three-dimensional models, the general features of the Cry48Aa model show a typical three-domain Cry toxin structure (Fig. 1). Domain I is predicted to contain the seven α-helical bundle, thought to be involved in pore formation, and domain III is modelled to have a β-sheet ‘jelly roll’ topology as seen in the Cry toxin structures in the PDB database. Domain II exhibits the anti-parallel β-sheets that are found in the other Cry toxin structures and the regions predicted to correspond to the exposed loops thought to be involved in receptor binding (Li *et al*., 1991; Grochulski *et al*., 1995; Galitsky *et al*., 2001; Morse *et al*., 2001; Boonserm *et al*., 2005). The domain II loops of the Cry48Aa model align to the region of Cry4Ba containing exposed loops and, as in Cry4Ba, these loops are smaller than in other toxins such as Cry3Aa and Cry1Aa. It has been shown that mutations in loop 3 of Cry4Ba can result in the introduction of toxicity to *Cx. quinquefasciatus* and *Cx. pipiens* larvae (Delécluse *et al*., 1993; Abdullah *et al*., 2006), towards which wild-type Cry4Ba shows no significant natural toxicity (Delécluse *et al*., 1993). Mutations in loops 1 and 2 also resulted in loss of toxicity towards *Aedes* and *Anopheles*, with no increase in toxicity towards *Culex*, confirming the importance of these regions in determination of specificity.

### Proteolytic processing of the toxin

Cry insecticidal toxins are produced during sporulation as parasporal crystals, which are solubilized in the insect gut and undergo proteolytic processing before receptor binding and membrane pore formation occurs. The major gut proteinases involved in Cry toxin processing are trypsin-like and chymotrypsin-like enzymes, while thermolysin-like and elastase-like enzymes have also been reported (Dai and Gill, 1993; Rukmini *et al*., 2000). Differential processing of the Cry pro-toxins by different larvae has also been shown to determine target insect toxicity (Haider *et al*., 1986; Haider and Ellar, 1987a, b).

**Fig. 2:**
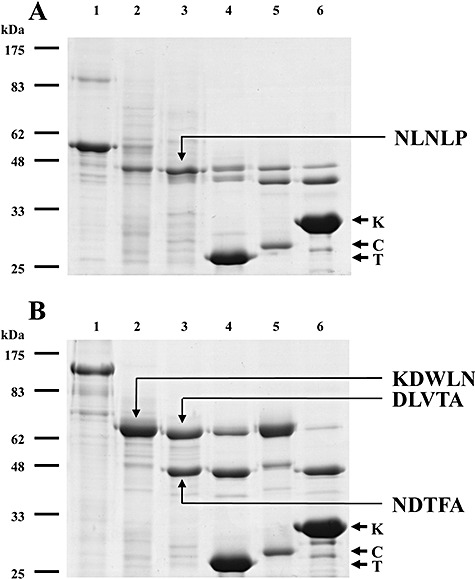
Digestion of Cry48 and Cry49 with gut extracts. Processing of Cry49Aa (A) and Cry48Aa (B) by *Ae. aegypti* gut extract (lane 2), *Cx. quinquefasciatus* gut extract (lane 3), trypsin (lane 4), chymotrypsin (lane 5) or proteinase K (lane 6). Undigested Cry protein is shown in lane 1. N-terminal sequences derived from the bands marked are given. Bands corresponding to the purified proteinases added in lanes 4–6 are marked T (trypsin), C (chymotrypsin) and K (proteinase K).

To determine whether the processing of the Cry48Aa/Cry49Aa toxin in *Ae. aegypti* and *Cx. quinquefasciatus* larvae might be responsible for the differential toxicity to these two mosquitoes, both Cry48Aa and Cry49Aa were incubated *in vitro* with larval gut extracts from these insects, as well as the enzymes trypsin, chymotrypsin and proteinase K (Fig. 2). Cry49Aa processing (Fig. 2A) produces bands of similar size on incubation with both gut extracts and with trypsin, chymotrypsin or proteinase K. N-terminal sequencing of the protein activated by *Cx. quinquefasciatus* gut extract identified the site of processing to be between F^48^ and N^49^, a chymotrypsin-like cleavage site.

In contrast to the apparently similar processing of Cry49Aa under all conditions, different patterns were seen (Fig. 2B) after incubation of Cry48Aa with *Ae. aegypti* gut extract and *Cx. quinquefasciatus* extracts. N-terminal sequencing of the product of Cry48Aa activated by *Ae. aegypti* extract identified a cleavage site between Y^35^ and K^36^, corresponding to a chymotrypsin-like cleavage on the carboxyl side of an aromatic amino acid. This is consistent with the similar protein bands observed after processing by *Ae. aegypti* gut extract and chymotrypsin (Fig. 2, lanes 2 and 5). Cry48Aa processed by *Cx. quinquefasciatus* gut extract yields two major products. Edman degradation revealed that the higher molecular mass product (∼60 kDa) was typical of chymotrypsin-like cleavage between Y^52^ and D^53^, while the lower molecular mass (∼46 kDa) product is a result of a trypsin-like cleavage between R^238^ and N^239^ (consistent with a similar banding pattern of trypsin incubation, Fig. 2B, lanes 2 and 5).

### Effect of activation on target range

As the processing of Cry48Aa by *Cx. quinquefasciatus* gut extracts produced smaller products than those from *Ae. aegypti* extracts, experiments were undertaken to determine whether differential processing of Cry48Aa and Cry49Aa in the two mosquito species might be responsible for the pattern of toxicity observed against the larvae of each species. Purified Cry48Aa and Cry49Aa were incubated with *Cx. quinquefasciatus* gut extract to allow processing as before. Following incubation, both samples were combined in a selective bioassay against *Ae. aegypti* larvae to test the possibility that the *Culex* activation of the proteins might confer toxicity to *Ae. aegypti.* Control bioassays were also prepared containing no toxin, and an un-processed Cry49Aa/Cry48Aa crystal protein mixture added directly to the bioassay. A bioassay using *Cx. quinquefasciatus* larvae, exposed to the Cry48Aa/Cry49Aa toxin pre-incubated with *Cx. quinquefasciatus* gut extract, was also prepared, as above, to confirm that the processed toxin retained toxicity when fed to susceptible larvae. No toxicity was observed against *Ae. aegypti* larvae, using either the activated Cry48Aa/Cry49Aa toxin or the crystal proteins. Bioassay of the activated toxin against *Cx. quinquefasciatus* larvae resulted in 100% mortality while no mortality was observed in the control bioassays containing no toxins.

## Discussion

The results presented here confirm our previous finding that both components of the Cry48Aa/Cry49Aa pair are necessary to cause toxicity in *Cx. quinquefasciatus* larvae. Synergism between the Bin toxins of *B. sphaericus*, which are related to Cry49Aa, and Cry toxins of *B. thuringiensis* has been reported previously (Wirth *et al*., 2004b). In our assays, the toxicity of a Cry49Aa/Cry4Aa combination was higher than predicted for this mixture against both susceptible and Cry4-resistant larvae, indicating possible synergy between the two proteins. Interactions of Cry49Aa with three-domain proteins other than Cry48Aa, thus appear to be a possibility.

The target range for the Cry48Aa/Cry49Aa toxin pair from *B. sphaericus* is apparently very narrowly limited to the genus *Culex*. The processing of the proteins by gut extracts was, thus, investigated as a possible source of toxicity differences with *Culex* and *Aedes* targets. Processing of Cry48Aa is significantly different with extracts from the two mosquitoes, with *Culex* extracts producing fragments akin to those produced by the cleavage of Cry4Aa and Cry4Ba by mosquito gut extracts or trypsin, which is thought to involve cleavage to a 60–68 kDa protein before further processing into two fragments of 46–48 and 16–18 kDa (Angsuthanasombat *et al*., 1991, 1992). Inspection of the homology model of Cry48Aa shows the processing site for the *Cx. quinquefasciatus* gut extract after R238 to lie in domain I, which comprises an α-helical bundle, known to be important for toxicity, involved in lysis of midgut epithelial cells by formation of pores. Processing at this site would result in cleavage in the long predicted inter-helical loop between the central helices 5 and 6, as shown in Fig. 1B. Processing between helices 5 and 6 is also known to occur in Cry4Aa and Cry4Ba, and it has been suggested that this may assist the Cry4Ba toxin to undergo a conformational change, facilitating the insertion of domain I into the membrane (Angsuthanasombat *et al*., 1993; Boonserm *et al*., 2005). However, removal of the inter-helical cleavage sites in Cry4Ba and Cry4Aa is not detrimental to toxicity, suggesting that processing at this site is not essential for the conformational change required for pore formation (Angsuthanasombat *et al*., 1993; Boonserm *et al*., 2004; Boonserm *et al*., 2006). Perhaps, consistent with this observation, differential processing of the Cry48Aa/Cry49Aa proteins in *Ae. aegypti* mosquitoes does not account for its non-toxicity to this insect as pre-processing with *Culex* extracts does not reveal *Aedes* toxicity. This may indicate that the specificity of this toxin is mediated by receptors in *Cx. quinquefasciatus* that may be absent from the *Ae. aegypti* larval gut. Previously described toxins do not necessarily kill all related insect species: for instance, Cry4A and Cry10A of *B. thuringiensis* ssp. *israelensis* are toxic to *Ae. aegypti*, but show negligible toxicity to *Chironomus tepperi* (Crickmore *et al*., 1995; Hughes *et al*., 2005); and the Bin toxin variants of *B. sphaericus* are highly active against *Cx. quinquefasciatus* but have little to no activity against *Ae. aegypti* (Berry *et al*., 1993), but most toxins are active against more than one species in a closely related group of insects. This makes the non-toxicity of the Cry48Aa/Cry49Aa combination against *Ae*. *aegypti* and *An. gambiae* particularly interesting.

*Culex quinquefasciatus* (more correctly *Cx. pipiens quinquefasciatus*) is one of four subspecies in the *Cx. pipiens* complex that also includes *Cx. pipiens pipiens.* The Cry48Aa/Cry49Aa toxin pair is able to overcome resistance in *Cx. quinquefasciatus* to the Bin toxin of *B. sphaericus* (Jones *et al*., 2007). Strains producing Cry48Aa/Cry49Aa, such as *B. sphaericus* IAB59, LP1G and 47-6B, can also overcome Bin resistance in *Cx. quinquefasciatus* larvae (Wirth *et al*., 2000; Pei *et al*., 2002; Yuan *et al*., 2003). These strains have also been observed to overcome resistance in *Cx. pipiens pipiens* larvae (Silva-Filha *et al*., 2004), indicating probable Cry48Aa/Cry49Aa toxicity to this related mosquito. Therefore, the Cry48Aa/Cry49Aa toxin should be considered as toxic to *Cx. pipiens* complex mosquitoes but, at least at present, this must be designated as the only known target for these proteins. This toxin therefore remains something of an enigma: an obligate binary toxin comprising members of two previously separate toxin families, capable of high-level toxicity in purified form but severely limited by low-level production of the Cry48Aa component *in vivo* and with a severely limited target range. It forms part of the arsenal of minor toxins of *B. sphaericus* that include the Mtx1, Mtx2, Mtx3 and predicted Mtx4 (Hu *et al*., 2008), vegetative toxins that may play minor roles in toxicity in the field but have significant potential for exploitation in strain improvement in this bacterium.

## Experimental procedures

### Strains and plasmids

Two recombinant *B. thuringiensis* ssp. *israelensis* strain 4Q7 derivatives containing either pSTABP135 or pHTP49 that direct production of Cry48Aa and Cry49Aa, respectively, were constructed in previous studies (Jones *et al*., 2007). These strains were grown in EMBRAPA medium (Monnerat *et al*., 2007) to > 98% sporulation (typically 48–72 h) as judged by phase contrast microscopy. At this time, they were harvested by centrifugation, washed in distilled water and lyophilized to produce powders for use in bioassays. For synergy studies, recombinant *B. thuringiensis* ssp. *israelensis* producing Cry4Aa (Delécluse *et al*., 1993) were also used.

### Toxicity assays

Qualitative bioassays against a range of insect targets were carried out as follows. Bioassays against the mosquitoes *Cx. quinquefasciatus*, *Ae. aegypti* and *An. gambiae* used 10 second or third instar larvae in 10 ml of dechlorinated tap water to which was added 100 μl of a sporulated culture of *B. sphaericus* or recombinant *B. thuringiensis.* Mortality was assessed after 24 and 48 h at 28°C. *Chironomus riparius* assays were performed as previously described (Partridge and Berry, 2002) except that 100 μl of sporulated cultures was added to 10 second instar larvae in 10 ml of distilled water containing sediments of homogenized Whatman 3Mm paper. The *Lepidoptera Ant. gemmatalis*, *S. frugiperda* and *P. xylostella* were assayed by the methods of Monnerat and colleagues (2007) with the addition of 150 μl of bacterial culture to artificial diet for *Ant. gemmatalis*, 30 μl culture to *S. frugiperda* diet and the use of 1:100 dilutions of cultures for the dipping of leaves for the *P. xylostella* assay. The coleopteran *Anth. grandis* was challenged with artificial diet containing 200 μl of sporulated culture using the procedure of Soares Martins and colleagues (2007). In all cases, *B. thuringiensis* recombinants expressing Cry48Aa and Cry49Aa were assayed both independently and in combination. When combinations were used, the quantities of culture listed above were added to each bioassay for each of the recombinant *B. thuringiensis* strains.

For synergy studies, Cry49Aa crystals were prepared from *B. thuringiensis* ssp. *israelensis* 4Q7::pHTP49 following sucrose density gradient centrifugation as previously described (Jones *et al*., 2007). The Cry4Aa stock was prepared from the sporulated, lyophilized bacterial powders of *B. thuringiensis* producing this toxin by suspending weighed powder in deionized water with 20–25 glass beads to promote homogenization. Different concentrations of the Cry4Aa suspensions were fed to groups of 20 early fourth instar larvae of *Cx. quinquefasciatus* strain Syn-P (Wirth *et al*., 2004a) in 250 ml plastic cups containing 100 ml of deionized water. Eight or more concentrations that produced mortality between 0% and 100% plus an untreated control were used for each dose–response test and replicated five times on five different days. Stocks of Cry49Aa powder were similarly prepared and showed no activity at 200 μg ml^−1^. Consequently, testing was limited to five replicates of 20 larvae per cup over five different days. Similar tests were conducted with Cry4Aa against the Cq4AB colony, with high resistance to Cry4Aa (Wirth and Georghiou, 1997). No control mortality was observed. Powders were combined by weight in a 3:1 ratio of Cry4Aa and Cry49Aa and tested against groups of 20 larvae at 2, 20 and 200 μg ml^−1^, and replicated three to five times on three to five different days. Susceptible and resistant mosquito colonies were tested concurrently using the same test materials and stock suspensions. Larvae received a small amount of food at 24 h and mortality was determined at 48 h. Cry4Aa data were analysed using Probit analysis (Finney, 1971). Probit analysis was not possible on all Cry49Aa data and on Cry4Aa data against Cq4AB; therefore, average mortality and standard deviation were calculated.

### Proteolytic processing of toxins

Extracts were prepared from the guts of fourth instar larvae of *Cx. quinquefasciatus* and *Ae. aegypti* as described by Thanabalu and colleagues (1992). Guts were dissected from 20 larvae and placed into ice-cold microfuge tubes containing 200 μl of PBS (140 mM NaCl, 2.7 mM KCl, 10 mM Na_2_HPO_4_, 1.8 mM KH_2_PO_4_, pH 7.3) following removal of the peritrophic membranes. The guts were homogenized in 1.5 ml microfuge tubes using a pestle that exactly fits the tube. The particulate material was then removed by centrifugation (17 000 *g*, 5 min), and the supernatant fraction was used fresh in proteolytic incubations.

Individual crystal proteins were purified from the recombinant *B. thuringiensis* on sucrose gradients (Jones *et al*., 2007), and ∼10 μg of each was solubilized in 50 mM NaOH for 1 h at 30°C and any insoluble material was removed by centrifugation (17 000 *g*, 5 min). The solution was then adjusted to 20 mM Tris-HCl, 150 mM NaCl, 2.5 mM CaCl_2_, pH 8.4 in a final volume of 100 μl. This solubilized toxin was treated with 10 μl of mosquito larval gut extract or 1 μg of proteolytic enzyme [trypsin, α-chymotrypsin or proteinase K (Sigma, Poole Dorset, UK)] and incubated at 30°C for 1 h. Control reactions were prepared in an identical manner to the test reactions, except that no gut extract or proteolytic enzymes were added prior to the incubation step. The digest products were then precipitated by the addition of trichloro acetic acid to 10% (w/v) final concentration and incubation on ice for 20 min. The precipitated protein was harvested by centrifugation (17 000 *g*, 15 min) and the pellet was washed with acetone, pre-cooled to −20°C. The samples were centrifuged (17 000 *g*, 5 min, 4°C), the supernatant discarded and the protein pellets allowed to air-dry before re-suspension in SDS-PAGE protein sample buffer and analysis of the digest products by SDS-PAGE in a 10% acrylamide gel, stained with Coomassie blue. The N-terminal sequences of processed products were determined by automated Edman degradation (Alta Bioscience, Birmingham, UK) of bands transferred to PVDF membrane from SDS-PAGE gels run with tricine in place of glycine in the running buffer.

### Molecular modelling of Cry48Aa

The homology-modelling server, swiss-model (Schwede *et al*., 2003), was used to generate a first-approximation three-dimensional model of Cry48Aa. This automated procedure involves submission of a primary amino acid sequence to the server, allowing selection of templates based on protein homology. An alignment of the submitted sequence and the templates is generated, followed by the building of the model backbone, side-chain modelling and energy minimization. Templates used in this case were Cry1Aa, Cry3Aa, Cry3Bb, Cry4Aa and Cry4Ba (ExPDB codes 1ciy, 1dLc, 1ji6A, 2c9kA and 1w99A respectively).
